# Call for Papers, Issue 1/2022: Digital Entrepreneurship: Opportunities, Challenges, and Impacts

**DOI:** 10.1007/s12599-020-00651-2

**Published:** 2020-05-13

**Authors:** Jörn H. Block, Kathryn Brohman, Dennis M. Steininger

**Affiliations:** 1 Faculty of Management, Trier University, Trier, Germany; 2 Witten Institute for Family Business (WIFU), Witten/Herdecke University, Witten, Germany; 3 Erasmus School of Economics, Erasmus University Rotterdam, Rotterdam, Netherlands; 4 Smith School of Business, Queen’s University, Kingston, ON Canada; 5 Faculty of Business and Economics, University of Augsburg, Augsburg, Germany

## Special Issue

Digital transformation creates new opportunities and is an important driver of novel and often disruptive innovation and value creation. Entrepreneurs are using digital technologies to finance innovation (Ahrens et al. 2019; Audretsch et al. 2016; Fisch 2019) or create new digital products, services, and business models. These activities are called digital entrepreneurship and related new ventures are commonly referred to as digital startups if their business logic fully relies on digital technologies for value creation and transfer (Berger et al. 2019; Steininger 2019). Such endeavors bring changes in entrepreneurial processes (e.g., opportunity recognition and pursuit), innovation, competences, control, financing, institutions, and ecosystems (Block et al. 2018b; Cram et al. 2016; Hoegen et al. 2018; Nambisan 2017; Sussan and Acs 2017; Veit et al. 2014). The analysis of innovation and its positive economic impacts have a long tradition in information systems as well as entrepreneurship research (Audretsch et al. 2006; Yoo et al. 2010). Today, digital entrepreneurship is recognized by many countries as a very important element of economic development and job creation (Block et al. 2018a). However, at the same time, it also challenges established industries and forces them to cope with these new developments and technologies that can have adverse consequences on the economy and society (Shen et al. 2018; Veit et al. 2014).

This special issue focuses on understanding digital entrepreneurship and its implications. Specifically, we encourage contributions that deal with the opportunities and challenges of digitalization for entrepreneurial endeavors, specificities of processes in digital startups and for developing digital business models, and the impacts on cities, regions, or countries.

## Invited Contributions

Submissions to this special issue are encouraged from all theoretical and methodological perspectives drawing from information systems, entrepreneurship, organizational behavior, strategic management, and others. Authors must clearly outline why their study is new and interesting for research and practice and how it relates to the theme of the special issue.

The following list of topics is neither exclusive nor exhaustive. We have structured them along three dimensions (Recker and von Briel 2019):

- Opportunities of Digital Entrepreneurship
  - Opportunity recognition via digital means
  - Novel use of digital technologies for entrepreneurial activities and venture development (e.g., digital tools for business model creation and evaluation, big data, AI)
  - Opportunities and management of digital methods of funding innovation and entrepreneurship (e.g., crowdfunding, ICOs)
  - New value creation and platform innovation through digital technologies
  - Corporate entrepreneurship and startups within established organizations to approach opportunities and challenges of digital transformation
  - Exploitation of missing regulation of digital entrepreneurship (e.g., Uber)

- Processes, Organization, and Challenges of Digital Entrepreneurship
  - Competence and team needs of (corporate) digital entrepreneurs
  - Digital entrepreneurial culture
  - The role of universities and entrepreneurial ecosystems for digital entrepreneurship
  - Impacts of digital technologies on entrepreneurial decision making
  - Use of Social media or crowdsourcing resources by entrepreneurs
  - Organization, organizational learning, and performance of digital startups
  - Management of digital business model innovation
  - Internationalization of digital startups
  - Alignment of digital capabilities and business models
  - Behavior of startups in digital platform ecosystems (e.g., App Entrepreneurs)
  - Development and management of new digital ecosystems by startups
  - Industry-specific classification schemes of startups or business models (e.g. sharing economy, blockchain, or AI business model types)
  - Changes in startup investment processes due to digitalization

- Impacts of Digital Entrepreneurship
  - Impacts of digital entrepreneurship on regions and countries (e.g., knowledge spill-over, innovation, job-creation, economic growth)
  - Investments in digital innovation and their economic or societal pay-offs
  - The dark side of new digital business models (e.g., negative impacts of sharing economy business models such as AirBnB or Uber on economy and society)
  - Influences of digital entrepreneurial and innovative activities on government policy or regulation

## Submission Guidelines

Please submit papers by 1 March 2021 at the latest via the journal’s online submission system (http://www.editorialmanager.com/buis/). Please observe the instructions regarding the format and size of contributions to Business & Information Systems Engineering (BISE). Papers should adhere to the submission general BISE author guidelines (http://www.bise-journal.com/author_guidelines).

All papers will be reviewed anonymously (double-blind process) by at least two referees with regard to relevance, originality, and research quality. In addition to the editors of the journal, including those of this special focus, distinguished international scholars will be involved in the review process.

## Paper Development Workshop

There will be a special issue paper development workshop at the 24th Annual Interdisciplinary Conference on Entrepreneurship, Innovation and SMEs (G-Forum) which will take place at KIT Karlsruhe, Germany, from 30 September to 2 October 2020 (https://www.fgf-ev.de/en/g-forum-conference-2020-karlsruhegermany/). In this paper development workshop, the special issue editors will provide feedback on early drafts or first paper versions. The submission to the G-Forum, however, is not a requirement for submitting to the special issue. Please Note: We are closely monitoring the current situation with regards to COVID-19 and the paper development workshop might therefore be canceled or moved to a digital setting.

## Schedule

- Deadline for submission: 1 March 2021
- Notification of the author 1: 3 May 2021
- Completion revision 1: 2 July 2021
- Notification of the author 2: 17 August 2021
- Completion Revision 2: 21 September 2021
